# Phenotypic and molecular characterization of antimicrobial resistance in *Trueperella pyogenes* strains isolated from bovine mastitis and metritis

**DOI:** 10.1186/s12866-019-1630-4

**Published:** 2019-12-27

**Authors:** Mobin Rezanejad, Sepideh Karimi, Hassan Momtaz

**Affiliations:** 1 Graduated of Veterinary Medicine, Faculty of Veterinary Medicine, Shahrekord Branch, Islamic Azad University, Shahrekord, Iran; 2 Department of Microbiology, Shahrekord Branch, Islamic Azad University, Shahrekord, Iran

**Keywords:** *Trueperella pyogenes*, Antibiotic resistance pattern, Virulence factors, Mammary infection, Uterine infection

## Abstract

**Background:**

*Trueperella pyogenes* is one of the most clinically imperative bacteria responsible for severe cases of mastitis and metritis, particularly in postpartum dairy cows. The bacterium has emergence of antibiotic resistance and virulence characters. The existing research was done to apprise the phenotypic and genotypic evaluation of antibiotic resistance and characterization of virulence factors in the *T. pyogenes* bacteria of bovine mastitis and metritis in postpartum cows.

**Methods:**

Two-hundred and twenty-six bovine mastitic milk and 172 uterine swabs were collected and transferred to laboratory. Samples were cultured and *T. pyogenes* isolates were subjected to disk diffusion and DNA extraction. Distribution of virulence and antibiotic resistance genes was studied by PCR.

**Results:**

Thirty-two out of 226 (14.15%) mastitic milk and forty-one out of 172 (23.83%) uterine swab samples were positive for *T. pyogenes*. Isolates of mastitic milk harbored the highest prevalence of resistance toward gentamicin (100%), penicillin (100%), ampicillin (90.62%), amoxicillin (87.50%) and trimethoprim-sulfamethoxazole (87.50%), while those of metritis harbored the highest prevalence of resistance toward ampicillin (100%), amoxicillin (100%), gentamicin (97.56%), penicillin (97.56%) and cefalexin (97.56%). *AacC*, *aadA1*, *aadA2* and *tetW* were the most generally perceived antibiotic resistance genes. All bacteria harbored *plo* (100%) and *fimA* (100%) virulence factors. *NanP*, *nanH*, *fimC* and *fimE* were also the most generally perceived virulence factors.

**Conclusions:**

All bacteria harbored *plo* and *fimA* virulence factors which showed that they can use as specific genetic markers with their important roles in pathogenicity of *T. pyogenes* bacteria. Phenotypic pattern of antibiotic resistance was confirmed by genotypic characterization of antibiotic resistance genes.

## Background

Mastitis is an inflammation of the mammary gland and udder tissue. It is a major endemic disease of dairy cattle. It usually occurs as an immune response to microbial invasion of the teat canal by variety of microbes sources present on the farm, and can also occur as a result of chemical, mechanical, or thermal injury to the cow’s udder [1]. Metritis is an inflammation of the uterus. It is mainly caused by infections, and usually is seen following calving complicated by dystocia, retained fetal membranes, twins or stillbirths in postpartum cows [2]. Mastitis and metritis are considered to be the most frequent and most costly production diseases in dairy herds of developing and developed countries. Bacteria are considered to be the prevalent causes of mastitis and metritis in dairy cows [1, 2].

*Trueperella pyogenes* (*T. pyogenes*), formerly *Arcanobacterium pyogenes* (*A. pyogenes*), is a well-recognized Gram-positive, non-motile, β-hemolytic, irregular/rod-shaped, non-spore-forming, ´coryneform´ bacterium that causes opportunistic pyogenic infections of economic importance in livestock. However, it rarely affects companion animals and humans [3]. *T. pyogenes* is a widespread opportunistic pathogen with boost presence in mucus layer of upper respiratory, urogenital and gastrointestinal tracts of livestock [3, 4]. In keeping with the ability of *T. pyogenes* to occur autogenous infection, the bacterium can act as an initial pathogen after occurrence of different trauma [3, 4]. *T. pyogenes* is a causative agent of metritis, abortion, mastitis, infertility and pneumonia in dairy herds [3–5]. It is also considered as one of the most routine causes of antibiotic resistance mastitis and metritis [5–7].

Numerous virulence markers are accompanied with the pathogenicity of infections caused by this bacterium. The most important virulence markers are *plo* (pyolysin encoding gene), *nanH* and *nanP* (neuraminidases encoding genes), *cbpA* (collagen-binding protein encoding gene) and diverse fimbrial markers (*fimA*, *fimC*, *fimG* and *fimE*) [8–10]. Additionally, *T. pyogenes* are mostly resist toward diverse kinds of antibiotic agents which encoded by presence of determined antibiotic resistance genes including *tetW* (tetracyclines resistance gene), *ermB* and *ermX* (macrolides resistance genes), *aacC* and *aadA1* and *aadA2* (aminoglycosides resistance genes) *dfr2a* (trimethoprim resistance genes), and *blaP1* (β-lactams resistance genes). *OrfE* is additional antibiotic resistance genes of this bacterium with indefinite function [10, 11].

Molecular and antibiotic resistance-based properties of *T. pyogenes* bacteria are relatively unknown in the cases of mastitis and metritis. Thus, the current study investigated phenotypic and genotypic characterization of antibiotic resistance and molecular characterization of *T. pyogenes* bacteria isolated from cases of mastitis and metritis in dairy cows in Iran.

### Methods

### Moral deliberation

The research was confirmed by the Moral Assembly of Research of the Faculty of Veterinary Medicine, Shahrekord Branch, Islamic Azad University, Shahrekord, Iran. Verification of this research project and the licenses related to sampling process were approved by the Prof. Hassan Momtaz (Approval Ref Number 251216892).

### Samples and study population

The present descriptive study was conducted during February 2017 and October 2018 at the Veterinary hospital of the Islamic Azad University, Shahrekord Branch, Shahrekord, Southwest Iran. Two-hundred and twenty-six raw milk samples and one-hundred and seventy-two uterine swab samples were randomly collected from postpartum cows with clinical mastitis and metritis, respectively. Clinical mastitis was determined using the California mastitis test (CMT) according to procedure described by Hoque et al. (2015) [12]. Presence of clinical metritis in cows were approved by an expert veterinary midwifery. All samples were transferred to Microbiology Research Center of the Islamic Azad University, Shahrekord Branch, shahrekord, Iran in cooler with ice-packs.

### Bacterial isolation and identification

Isolation and identification of *T. pyogenes* bacteria were performed using the technique described beforehand [7]. For this goal, brain heart infusion agar (BHI, Merck, Germany) supplemented with 5% sheep blood and MacConkey agar (Merck, Germany) (incubated on aerobic and anaerobic circumstances for 48 h at 37 °C) were used for initial enrichment. *T. pyogenes* bacteria were identified by diverse biochemical tests such as CAMP tests (*Staphylococcus aureus* was used as indicator), urease catalase, nitrate reduction, oxidase, gelatin and esculin hydrolysis and finally of lactose, glucose, mannitol, maltose, xylose and sucrose fermentation tests [7].

### Phenotypic analysis of antibiotic resistance

Guidelines of the Clinical and Laboratory Standards Institute (CLSI) [13] according to Kirby-Bauer disk diffusion method was used for this goal. Mueller–Hinton agar (Merck, Germany) supplemented with 5% sheep blood was used for this goal. Pattern of antibiotic resistance of *T. pyogenes* bacteria was studied toward amoxicillin (25 μg), ampicillin (10 μg), azithromycin (15 μg), cefalexin (30 μg), ciprofloxacin (5 μg), enrofloxacin (5 μg), erythromycin (15 μg), gentamicin (10 μg), penicillin (10 units), rifampicin (5 μg), streptomycin (10 μg), sulfamethoxazole (25 μg), tetracycline (30 μg), trimethoprim- lincomycin (2 μg) and tylosin (30 μg) antibiotic disks (Oxoid, UK). *T. pyogenes* ATCC 19411 was used as quality control organism. Procedure was performed according to CLSI [13].

### DNA extraction

*T. pyogenes* bacteria were sub-cultured on nutrient broth media (NB, Merck, Germany) and further incubated for 48 h at 37 °C. Genomic DNA was extracted from bacterial colonies using the DNA extraction kit (Thermo Fisher Scientific, St. Leon-Rot, Germany) based on the guidelines of the factory. Purity, quality and quantity of extracted DNA were measured using Nanodrop device (NanoDrop, Thermo Scientific, Waltham, MA, USA), gel electrophoresis and spectrophotometer.

### Genotypic analysis of antibiotic resistance and virulence factors

Table 1 signifies the PCR circumstances used for amplification of antibiotic resistance genes [14–17]. Table 2 signifies the PCR circumstances used for amplification of virulence factors [18, 19]. A programmable DNA thermo-cycler (Eppendorf Mastercycler 5330, Eppendorf-Nethel-Hinz GmbH, Hamburg, Germany) was used in all PCR reactions. Gel electrophoresis (120 V/208 mA) in 2.5% agarose gel was applied for study the amplification of targeted genes. The UVI doc gel documentation systems (Grade GB004, Jencons PLC, London, UK) was applied for gel visualization.

**Table 1:** List of primers and PCR conditions used for amplification of antibiotic resistance genes in the *T. pyogenes* bacteria isolated from samples of mastitis and metritis [14–18]

Target gene	Primer sequence (5′-3′)	PCR product (bp)	PCR programs	PCR volume (50 μL)
Class 1 intI	F: CCTCCCGCACGATGATC	280	1 cycle:	5 μL PCR buffer 10X
	R: TCCACGCATCGTCAGGC		95 ^0C^ ------------ 3 min	2 mM Mgcl_2_
Class 1 cassette	F: GGCATCCAAGCAGCAAG R: AAGCAGACTTGACCTGA	Unpredictable	35 cycle: 94 ^0C^ ------------ 60 s	150 μM dNTP (Fermentas) 0.75 μM of each primers F & R
			58 ^0C^ ------------ 60 s 72 ^0C^ ------------ 60 s 1 cycle: 72 ^0C^ ------------ 5 min	1.5 U Taq DNA polymerase (Fermentas) 1 μL DNA template
Class 2 intI	F: TTATTGCTGGGATTAGGC R: ACGGCTACCCTCTGTTATC	233	1 cycle: 94 ^0C^ ------------ 4 min. 35 cycle: 94 ^0C^ ------------ 60 s 56 ^0C^ ------------ 60 s 72 ^0C^ ------------ 45 s 1 cycle: 72 ^0C^ ------------ 7 min	5 μL PCR buffer 10X 2 mM Mgcl_2_ 150 μM dNTP (Fermentas) 0.75 μM of each primers F & R 1 U Taq DNA polymerase (Fermentas) 1.5 μL DNA template
tetW	F: GACAACGAGAACGGACACTATG R: CGCAATAGCCAGCAATGAACGC	1843	1 cycle: 94 ^0C^ ------------ 2 min. 35 cycle: 94 ^0C^ ------------ 60 s 55 ^0C^ ------------ 60 s 72 ^0C^ ------------ 60 min 1 cycle: 72 ^0C^ ------------ 5 min	5 μL PCR buffer 10X 2 mM Mgcl_2_ 150 μM dNTP (Fermentas) 0.75 μM of each primers F & R 1.5 U Taq DNA polymerase (Fermentas) 3 μL DNA template
ermX	F:GTTGCGCTCTAACCGCTAAGGC R:CCATGGGGACCACTGAGCCGTC	571/657	1 cycle: 94 ^0C^ ------------ 2 min. 35 cycle: 94 ^0C^ ------------ 60 s 55 ^0C^ ------------ 60 s 72 ^0C^ ------------ 60 min 1 cycle: 72 ^0C^ ------------ 6 min	5 μL PCR buffer 10X 2 mM Mgcl_2_ 150 μM dNTP (Fermentas) 0.75 μM of each primers F & R 1.5 U Taq DNA polymerase (Fermentas) 3 μL DNA template
ermB	F: GAAATTGGAACAGGTAAAGG R: TTTACTTTTGGTTTAGGATG	404	1 cycle: 94 ^0C^ ------------ 60 s. 35 cycle: 94 ^0C^ ------------ 60 s 53 ^0C^ ------------ 60 s 72 ^0C^ ------------ 60 s 1 cycle: 72 ^0C^ ------------ 5 min	5 μL PCR buffer 10X 2 mM Mgcl_2_ 150 μM dNTP (Fermentas) 0.75 μM of each primers F & R 1.5 U Taq DNA polymerase (Fermentas) 3 μL DNA template

**Table 2:** List of primers and PCR conditions used for amplification of virulence factors in the *T. pyogenes* bacteria isolated from samples of mastitis and metritis [19, 20]

Target gene	Primer sequence (5′-3′)	PCR product (bp)	PCR programs	PCR volume (50 μL)
nanH	F: CGCTAGTGCTGTAGCGTTGTTAAGT R: CCGAGGAGTTTTGACTGACTTTGT	781	1 cycle: 94 ^0C^ ------------ 3 min. 35 cycle: 94 ^0C^ ------------ 60 s 60 ^0C^ ------------ 60 s 72 ^0C^ ------------ 3 min 1 cycle: 72 ^0C^ ------------ 7 min	5 μL PCR buffer 10X 1.5 mM Mgcl_2_ 200 μM dNTP (Fermentas) 0.5 μM of each primers F & R 1.25 U Taq DNA polymerase (Fermentas) 2.5 μL DNA template
nanP	F: TTGAGCGTACGCAGCTCTTC R: CCACGAAATCGGCCTTATTG	150		
cbpA	F: GCAGGGTTGGTGAAAGAGTTTACT R: GCTTGATATAACCTTCAGAATTTGCA	124		
fimC	F: TGTCGAAGGTGACGTTCTTCG R: CAAGGTCACCGAGACTGCTGG	843		
plo	F: GGCCCGAATGTCACCGC R: AACTCCGCCTCTAGCGC	270	1 cycle: 94 ^0C^ ------------ 2 min 35 cycle: 94 ^0C^ ------------ 60 s 55 ^0C^ ------------ 60 s 72 ^0C^ ------------ 60 s 1 cycle: 72 ^0C^ ------------ 5 min	5 μL PCR buffer 10X 2 mM Mgcl_2_ 150 μM dNTP (Fermentas) 0.75 μM of each primers F & R 1.5 U Taq DNA polymerase (Fermentas) 3 μL DNA template
fimA	F: CACTACGCTCACCATTCACAAG R: GCTGTAATCCGCTTTGTCTGTG	605	1 cycle: 94 ^0C^ ------------ 3 min. 35 cycle: 94 ^0C^ ------------ 60 s 57 ^0C^ ------------ 60 s 72 ^0C^ ------------ 3 min 1 cycle: 72 ^0C^ ------------ 7 min	5 μL PCR buffer 10X 2 mM Mgcl_2_ 150 μM dNTP (Fermentas) 0.75 μM of each primers F & R 1.5 U Taq DNA polymerase (Fermentas) 3 μL DNA template
fimG	F: ACG CTT CAG AAG GTC ACC AGG R: ATC TTG ATC TGC CCC CAT GCG	929		
fimE	F: GCCCAGGACCGAGAGCGAGGGC R: GCCTTCACAAATAACAGCAACC	775	1 cycle: 94 ^0C^ ------------ 3 min. 35 cycle: 94 ^0C^ ------------ 60 s 55 ^0C^ ------------ 60 s 72 ^0C^ ------------ 3 min 1 cycle: 72 ^0C^ ------------ 7 min	5 μL PCR buffer 10X 2 mM Mgcl_2_ 150 μM dNTP (Fermentas) 0.75 μM of each primers F & R 1.5 U Taq DNA polymerase (Fermentas) 3 μL DNA template

### Statistical analysis

Data were classified using the Microsoft Office Excel software. SPSS 21.0 statistical software (SPSS Inc., Chicago, IL, USA) and Chi-square and Fisher’s exact two-tailed tests were applied for statistical analysis of data. *P* value < 0.05 was considered as statistical significant level.

### Results

A total of 226 bovine mastitic milk and 172 bovine uterine swab samples were studied for prevalence of *T. pyogenes* bacteria, as well as phenotypic and genotypic evaluation of antibiotic resistance and distribution of virulence factors. Thirty-two out of 226 (14.15%) mastitic milk and forty-one out of 172 (23.83%) uterine swab of cows with metritis were positive for *T. pyogenes*. Statistically momentous variance was found for the prevalence of *T. pyogenes* between mastitis and metritis samples (*P* < 0.05).

Table 3 signifies the phenotypic pattern of antibiotic resistance amongst the *T. pyogenes* bacteria isolated from samples of mastitis and metritis in postpartum cows. *T. pyogenes* bacteria isolated from the mastitic milk samples harbored the highest prevalence of resistance toward gentamicin (100%), penicillin (100%), ampicillin (90.62%), amoxicillin (87.50%), trimethoprim-sulfamethoxazole (87.50%), cefalexin (84.37%) and streptomycin (81.25%) antibiotic agents. *T. pyogenes* bacteria isolated from the uterine swabs taken from cows with metritis harbored the highest prevalence of resistance toward ampicillin (100%), amoxicillin (100%), gentamicin (97.56%), penicillin (97.56%) and cefalexin (97.56%) antibiotic agents. Prevalence of resistance toward azithromycin antibiotic agents were low in both studied groups.

**Table 3:** Phenotypic pattern of antibiotic resistance amongst the *T. pyogenes* bacteria isolated from samples of mastitis and metritis in cow

Types of samples (N. positive)	Antibiotic resistance pattern (%)														
	Rifampicin	Streptomycin	Azithromycin	Tylosin	Cefalexin	Trimethoprim-sulfamethoxazole	Lincomycin	Tetracycline	Ciprofloxacin	Enrofloxacin	Penicillin	Amoxicillin	Ampicillin	Erythromycin	Gentamicin
Mastitis (32)	19 (59.37)	26 (81.25)	12 (37.50)	20 (62.50)	27 (84.37)	28 (87.50)	14 (43.75)	17 (53.12)	17 (53.12)	19 (59.37)	32 (100)	28 (87.50)	29 (90.62)	17 (53.12)	32 (100)
Metritis (41)	28 (68.29)	23 (56.09)	19 (46.34)	26 (63.41)	40 (97.56)	29 (70.73)	24 (58.53)	20 (48.78)	29 (70.73)	30 (73.17)	40 (97.56)	41 (100)	41 (100)	16 (39.02)	40 (97.56)

Table 4 signifies the genotypic pattern of antibiotic resistance amongst the *T. pyogenes* bacteria isolated from samples of mastitis and metritis in postpartum cows. *AacC* (87.50%), *aadA1* (81.25%), *aadA2* (56.25%) and *tetW* (56.25%) were the most generally perceived antibiotic resistance genes amongst the *T. pyogenes* bacteria isolated from mastitic milk samples. *AacC* (53.65%), *aadA1* (58.78%), *orfE* (48.78%) and *tetW* (48.78%) were the most generally perceived antibiotic resistance genes amongst the *T. pyogenes* bacteria isolated from uterine swab samples taken from cows with metritis. Statistically momentous variance was found between type of samples and distribution of antibiotic resistance genes of *T. pyogenes* bacteria (*P* < 0.05). Additionally, Statistically momentous variance was found between prevalence of *ermB* and *ermX* (*P* < 0.05) and *aadA1* and *aadA2* (*P* < 0.05) antibiotic resistance genes.

**Table 4:** Genotypic pattern of antibiotic resistance amongst the *T. pyogenes* bacteria isolated from samples of mastitis and metritis in cow

Types of samples (N. positive)	Antibiotic resistance genes (%)								
	*tetW*	*ermB*	*ermX*	*orfE*	*dfr2a*	*blaP1*	*aadA2*	*aadA1*	*aacC*
Mastitis (32)	18 (56.25)	10 (31.25)	14 (43.75)	16 (50)	12 (37.50)	14 (43.75)	18 (56.25)	26 (81.25)	28 (87.50)
Metritis (41)	20 (48.78)	14 (34.14)	18 (43.90)	20 (48.78)	18 (43.90)	19 (46.34)	17 (41.46)	20 (48.78)	22 (53.65)

Table 5 signifies the distribution of virulence factors amongst the *T. pyogenes* bacteria isolated from samples of mastitis and metritis in postpartum cows. *Plo* (100%), *fimA* (100%), *nanP* (84.37%), *fimC* (87.12%) and *fimE* (75%) were the most generally perceived virulence factors amongst the *T. pyogenes* bacteria isolated from mastitic milk samples. *Plo* (100%), *fimA* (100%), *nanH* (97.56%), *nanP* (92.68%), *fimE* (92.68%), *fimC* (78.04%) and *fimG* (73.17%) were the most generally perceived virulence factors amongst the *T. pyogenes* bacteria isolated from uterine swab samples collected from cows with metritis. Statistically momentous variance was found between type of samples and distribution of virulence factors of *T. pyogenes* bacteria (*P* < 0.05). Additionally, Statistically momentous variance was found between prevalence of *fimC* and *fimG* (*P* < 0.05), *nanP* and *nanH* (*P* < 0.05) and *fimE* and *fimG* (*P* < 0.05) virulence factors.

**Table 5:** Distribution of virulence factors amongst the *T. pyogenes* bacteria isolated from samples of mastitis and metritis in cow

Types of samples (N. positive)	Virulence factors (%)							
	*plo*	*fimG*	*fimE*	*fimC*	*fimA*	*cbpA*	*nanP*	*nanH*
Mastitis (32)	32 (100)	2 (6.25)	24 (75)	25 (78.12)	32 (100)	6 (18.75)	27 (84.37)	20 (62.50)
Metritis (41)	41 (100)	30 (73.17)	38 (92.68)	32 (78.04)	41 (100)	26 (63.41)	38 (92.68)	40 (97.56)

### Discussion

Prevalence of *T. pyogenes* bacteria in samples of mastitis and metritis in postpartum cows were 14.15 and 23.83%, respectively. Epidemiological investigations revealed that bovine intramammary infections caused by *T. pyogenes* are associated with the highest somatic cell count (SCC) in milk and significant losses in milk yield, as well as high percentages of nonfunctional quarters [20, 21]. Furthermore, the efficacy of intramammary therapy of mastitis caused by this bacterium is rather low [20, 21]. *T. pyogenes* was the most important bacterial risk factor for clinical endometritis, but not for subclinical endometritis. *T. pyogenes* bovine metritis is characterized by fever, a fetid vulvar discharge, excessive fluid discharge and lacking tone of uterus and off-feed and depression. It is occasionally acute disease that can be life threatening [22, 23]. Tamai et al. (2018) [24] described that 65 *T. pyogenes* bacteria (32.50%) were isolated from 200 collected samples, 16 (24.60%) of which were isolated as pure cultures, as opposed the other 49 isolates (75.40%) were obtained from mixed cultures which was higher than our stated findings. Prevalence of *T. pyogenes* in mastitic milk samples collected from Iran was 36.50% [25] which was higher than our stated findings. A cross-section study in Brazil, which was conducted through 2002 to 2012 revealed that mastitis (45.10%), abscesses (18.00%), pneumonia (11.10%), and lymphadenitis (9.00%) were the most common clinical manifestations occurred due to the *T. pyogenes* [3]. Furthermore, *T. pyogenes* was also isolated from other miscellaneous clinical specimens from cases of septicemia, encephalitis, pyometra, prostatitis, orchitis, seminal vesiculitis, pericarditis, and omphalitis [3]. High prevalence of *T. pyogenes* bacteria in the cases of mastitis and metritis in postpartum cows has been described from China [26], Germany [27], Poland [28] and Egypt [29].

The development of antimicrobial resistance by bacteria is a global problem. Overuse of antimicrobial agents, incomplete treatment, wrong choice of medication and transfer of antibiotic resistance genes among bacteria are the main reasons for the increase in antibiotic resistance. Findings of the current investigation revealed that the phenotypic pattern of the antibiotic resistance was confirmed by the genotypic pattern. Otherwise, higher prevalence of *tetW*, *ermB* and *ermX*, *aacC* and *aadA1* and *aadA2*, *dfr2a*, and *blaP1* antibiotic resistance genes were found in tetracycline, macrolides, aminoglycosides, trimethoprim, and β-lactams-resistant *T. pyogenes* bacteria. We found that *T. pyogenes* bacteria isolated from the mastitic milk and also uterine swabs taken from cows with metritis harbored the highest prevalence of resistance toward gentamicin, penicillin, ampicillin, amoxicillin and cefalexin antibiotic agents. High prevalence of antibiotic resistance described in the existing research is may be due to the unofficial and unselective antibiotic prescription in Iranian veterinary hospitals. Boost incidence of resistance of *T. pyogenes* bacteria toward gentamicin, penicillin, ampicillin, amoxicillin and cefalexin antibiotic agents was also described from Lithuania [30], Senegal [31] and Brazil [32]. Moreover, boost incidence of *tetW*, *ermB* and *ermX*, *aacC* and *aadA1* and *aadA2*, *dfr2a*, and *blaP1* antibiotic resistance genes in the *T. pyogenes* bacteria was also described from China [9], Iran [25] and Poland [33]. Momtaz et al. (2016) [25] described that *T. pyogenes* bacteria isolated from bovine mastitic milk samples harbored the highest prevalence of resistance toward tetracycline (97.80%), gentamicin (86.90%),streptomycin (84.80%), penicillin (69.60%), erythromycin (63.00%), trimethoprim-sulfamethoxazole (39.10%), enrofloxacin (23.90%) and ciprofloxacin (19.60%) which was similar to our findings. Similarly, more than 50% of the isolates recovered from bovine metritis samples were simultaneously resist toward penicillin and ampicillin [34]. Rife prescription of tylosin and tetracyclines as therapeutic and growth stimulating agents have resulted in high occurrence of resistant *T. pyogenes* [24, 33, 34]. Askari et al. (2018) [35] described the boost incidence of resistance of *T. pyogenes* bacteria toward gentamycin, tetracycline, cefotaxime, ciprofloxacin, erythromycin, vancomycin, streptomycin, ampicillin and doxycycline antibiotic agents. Dong et al. (2017) [36] described that *strB*, *aph*A1, *sul1* and *aac (6′)-Ib* were the most generally perceived antibiotic resistance genes amongst the *T. pyogenes* bacteria isolated from animal clinical infections. Zhao et al. (2011) [37] described that the incidence of *aacC*, *aadA1*, *aadA2*, *blaP1*, *dfr2a* and *orfE* antibiotic resistance genes were 3.60, 32.10, 21.40, 28.60, 21.40 and 28.60%, respectively which was similar to our findings. They also showed that the incidence of aminoglycoside-resistance markers was 57.10%. The tetracycline resistant phenotype is principally associated with *tetW* gene, which is responsible for resistance in a wide range of bacteria recovered from both human and animal [15]. It has been shown that *ermX* and *ermB* genes encoded *rRNA* methylases which associated with resistance to MLS_B_ antibiotics [33]. Additionally, the incidence of *ermX* is much higher than of *ermB* amongst the tylosin-resistant *T. pyogenes* [33]. Aminoglycoside-resistance determinants (*aacC*, *aadA1* and *aadA2*), β-lactam-resistance determinant (*blaP1*) and trimethoprim-resistance determinant (*dfr2a*) were prevalent among *A. pyogenes* isolates of other clinical infections [9, 15, 33, 37].

Our findings showed that all of the *T. pyogenes* bacteria harbored *plo* (100%) and *fimA* (100%) virulence factors. Prevalence of *nanH* was also near to 100% amongst the *T. pyogenes* bacteria isolated from uterine swabs of cows with metritis. Additionally, *nanP*, *fimC* and *fimE* were the most generally perceived virulence factors amongst the *T. pyogenes* bacteria. Higher prevalence of *plo* and *nanH* virulence genes was also detected in previous researches [8–10, 24, 25, 28]. However, low distribution of *nanP*, *cbpA* and fimbrial genes were described [8–10, 23, 24, 27]. *fimA* gene was detected in nearly 94% of *T. pyogenes* isolates which originated from animal sources [38] and in 76.40% isolates recovered from metritis in dairy cows [10, 19]. We detected the *fimA* gene in all *T. pyogenes* isolates of bovine mastitis and metritis origins, which was similar to results stated by Hijazin et al. (2011) [27]. Thus, *plo* and *fimA* virulence factors may have the higher importance in the pathogenesis of the mastitis and metritis diseases caused by *T. pyogenes*. These genes may also be good genetic markers in detection of *T. pyogenes* bacteria in clinical samples. Pyolysin is a major virulence factor responsible for lysis of host cells.

Most of the isolates recovered from milk (75%) and those of metritis (92.68%) were positive *fimE* gene which was similar to findings of Silva et al. (2008) [19]. *FimG* gene had the higher prevalence in *T. pyogenes* bacteria isolated from the cases of metritis (73.17%) than those of mastitis (6.25%). The *fimG* genewas also more prevalent among isolates of bovine metritis (50–67%) [10, 19], compared to mastitis (18%). Thus, the *fimG* gene may be predominant among *T. pyogenes* bacteria isolated from the cases of metritis.

The collagen-binding protein gene (*CbpA*) was detected in 18.75 and 63.41% isolates recovered from clinical mastitis and metritis, respectively. *CbpA* was also detected in 21% isolates recovered from clinical mastitis [33]. Prevalence of *cbpA* gene in *T. pyogenes* strains isolated from bovine clinical infection [39] and uterine secretion [10, 19] were 48.9 and 100%, respectively. Higher prevalence of *cbpA* gene was described in the cases of metritis [24, 40].

We also found that *T. pyogenes* elaborates at least two neuraminidases encoded by nanH and nanP proteins. Otherwise, prevalence of nanP protein in the cases of mastitis and metritis was 84.37 and 92.68%, respectively. Moreover, prevalence of nanH protein in the cases of mastitis and metritis was 62.50 and 97.56%, respectively. Two neuraminidases, nanH and nanP, are proteins undoubtedly underwriting to the colonization of host tissue. Previous works [41, 42] showed that all investigated *T. pyogenes* isolates recovered from diverse kinds of infections were positive for nanH protein and 64.2% of them harbored nanP protein. Both nanH and nanP proteins were detected in majority of *T. pyogenes* bacteria isolated from bovine clinical samples studied in Iran [25], USA [43], Portugal [10] and Brazil [44]. Reversely, nanH protein was not detected in the bovine clinical samples studied by Hijazin et al. (2011) [27]and Zastempowska et al. (2012) [33]. A comparison of these results may lead us to conclude that neuraminidases, especially nanH, may not be the main virulence marker occupied in the pathogenesis of bovine clinical infections. Momtaz et al. (2016) [25] described that the prevalence of *plo*, *fimA*, *fimC*, *fimG*, *nanP*, *cbpA* and *nanH* virulence markers amongst the *T. pyogenes* bacteria isolated from bovine mastitic milk samples were 100, 100, 84.70, 26, 21.70, 17.30 and 15.20%, respectively. Silva et al. (2008) [20] described that the prevalence of *plo*, *nanH*, *nanP*, *cbpA*, *fimA*, *fimC*, *fimE* and *fimG* virulence factors amongst the *T. pyogenes* bacteria isolated from bovine clinical metritis were 100,100, 100, 100, 100, 67, 98 and 67%, respectively which was also similar to our findings. Similar patterns for virulence factors were also described by Rzewuska et al. (2012) [28].

## Conclusions

High prevalence of resistance toward gentamicin, penicillin, ampicillin, amoxicillin and cefalexin antibiotic agents and high prevalence of *aacC*, *aadA1*, *aadA2* and *tetW* antibiotic resistance genes in the *T. pyogenes* bacteria were the most important findings of our study. Furthermore, *T. pyogenes* bacteria harbored certain putative virulence factors, especially *plo*, *fimA*, *nanP*, *nanH*, *fimC* and *fimE* which showed their high pathogenicity. All of the *T. pyogenes* bacteria harbored *plo* and *fimA* virulence factors which showed that they can use as specific genetic markers for detection of pathogenic *T. pyogenes* bacteria in the cases of mastitis and metritis in postpartum cows. Phenotypic pattern of antibiotic resistance was approved by the genotypic characterization of antibiotic resistance genes. The presence of detected gene cassettes in bacterial isolates indicates that integrons may play an important role in the dissemination of antimicrobial resistance. However, further investigation are needed to found the exit relations between distribution of virulence factors and antibiotic resistance genes and other epidemiological aspects of virulent and resistant *T. pyogenes* bacteria in the cases of mastitis and metritis in postpartum cows.

## Data Availability

All data analyzed during this study are included in this published article.

## Abbreviations

CLSI: Clinical and Laboratory Standards Institute.
*T*: *pyogenes*: *Trueperella pyogenes*
